# Freeform 3D Ice Printing (3D‐ICE) at the Micro Scale

**DOI:** 10.1002/advs.202201566

**Published:** 2022-07-06

**Authors:** Akash Garg, Saigopalakrishna S. Yerneni, Phil Campbell, Philip R. LeDuc, O. Burak Ozdoganlar

**Affiliations:** ^1^ Department of Mechanical Engineering Carnegie Mellon University Pittsburgh PA 15232 USA; ^2^ Department of Biomedical Engineering Carnegie Mellon University Pittsburgh PA 15232 USA; ^3^ Departments of Mechanical Engineering Biomedical Engineering Biological Sciences and Computational Biology Carnegie Mellon University Pittsburgh PA 15232 USA; ^4^ Departments of Mechanical Engineering Biomedical Engineering and Material Science and Engineering Carnegie Mellon University Pittsburgh PA 15232 USA

**Keywords:** cryogenic printing, free‐form printing, hierarchical channels, ice printing, ice templating, micro‐casting, out‐of‐plane printing

## Abstract

Water is one of the most important elements for life on earth. Water's rapid phase‐change ability along with its environmental and biological compatibility also makes it a unique structural material for 3D printing of ice structures reproducibly and accurately. This work introduces the freeform 3D ice printing (3D‐ICE) process for high‐speed and reproducible fabrication of ice structures with micro‐scale resolution. Drop‐on‐demand deposition of water onto a −35 °C platform rapidly transforms water into ice. The dimension and geometry of the structures are critically controlled by droplet ejection frequency modulation and stage motions. The freeform approach obviates layer‐by‐layer construction and support structures, even for overhang geometries. Complex and overhang geometries, branched hierarchical structures with smooth transitions, circular cross‐sections, smooth surfaces, and micro‐scale features (as small as 50 µm) are demonstrated. As a sample application, the ice templates are used as sacrificial geometries to produce resin parts with well‐defined internal features. This approach could bring exciting opportunities for microfluidics, biomedical devices, soft electronics, and art.

## Graphical abstract

A freeform 3D printing process (3D‐ICE) is presented to print 3D complex microscale ice structures rapidly and reproducibly with circular cross‐sections, smooth walls, hierarchical branched geometries, and overhang features. As a sample application, the printed ice templates are used as sacrificial geometries to produce resin parts with well‐defined internal features.

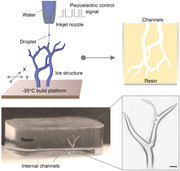

## 1 Introduction

Water is the primary building block of any living organism and the most abundant substance on the earth's surface. Although water has been used as a resource for many applications, the use of ice as a structural material is relatively uncommon. However, the simple and rapid phase transition of liquid water to ice and ice to liquid water or water vapor can provide exciting opportunities to use water as an environmentally friendly and biocompatible structural material for many advanced applications. In particular, ice can be used to create sacrificial templates that later form conduit and other open features inside fabricated parts. Taking advantage of the intriguing benefits of water as a structural material requires developing reproducible techniques for fabricating complex, multi‐scale, and high‐resolution structures of ice. This work introduces a freeform 3D printing process (3D‐ICE) for fabricating ice structures with micro‐scale resolution that can also be used for reverse molding.

We follow the 3D droplet‐on‐demand (inkjet) printing approach with water as the printing ink. Inkjet printing is a non‐contact, maskless, scalable, and high‐resolution process that has found extensive applications in the electronics, pharmaceutical, and biomedical industries.^[^ ^1^ ^]^ Originally developed as a 2D process for printing graphics, inkjet printing has since evolved into a 3D printing process for diverse applications, including patterning of wax,^[^ ^2^ ^]^ binders for ceramic 3D printing,^[^ ^3^ ^]^ and biological inks for tissue engineering. In tissue engineering, 3D inkjet printing has enabled the precision placement of cells in scaffolds and facilitated co‐printing of cells and hydrogels.^[^ ^4^ ^]^ 3D inkjet printing of polymers involves the controlled deposition of liquid ink in the form of droplets.^[^ ^5^ ^]^ The deposited ink is then solidified, commonly through ultra‐violet (UV) curing or solvent evaporation, to create the final structures. Generally, 3D inkjet printing is performed through layer‐by‐layer deposition: a thin layer of liquid polymer is deposited onto a platform in a specified pattern and then cured/dried before another layer is deposited. This layer‐by‐layer process significantly limits processing speed and print resolution and necessitates incorporating support structures in fabricating complex geometries, including undercuts and overhangs.^[^ ^6^ ^]^

Recently, a few works have demonstrated droplet‐based inkjet printing of water to print micro‐scale ice templates.^[^ ^7^, ^8^, ^9^ ^]^ The inkjet ice printing method was first introduced by Zheng et al.^[^ ^7^, ^8^ ^]^ The authors used a voxel‐based ice printing method, where a voxel was frozen before the next voxel was deposited above it. Each independent voxel can either be an individual droplet or an agglomeration of multiple droplets deposited by using a high‐frequency burst, referred to as “the trigger mode.” Using this approach, Zheng et al., demonstrated the fabrication of various ice structures, including straight and overhung geometries with cross‐sectional diameters equal to the diameter of individual deposited voxels, as well as interesting and challenging geometries such as the letters in the alphabet. However, the voxel‐based approach poses challenges in the fabrication of structures with smooth surfaces and hierarchical geometries with smooth transitions, especially when the cross‐sectional dimensions are different from the droplet size. In another recent work,^[^ ^9^ ^]^ researchers used a multi‐nozzle inkjet printing system to fabricate ice structures. A layer‐by‐layer approach was used, enabling 2.5D (extruded geometry) ice structures to be fabricated.

Other works have explored the 3D printing of diverse materials under freezing temperatures at larger size scales. Rapid‐freeze prototyping was used to fabricate centimeter‐scale ice sculptures using a steady stream of water deposited on a cold build platform.^[^ ^10^, ^11^ ^]^ Although the continuous stream enabled high‐speed printing of large‐scale ice structures, it impeded the ability to print small or precise features. Further, support‐free printing could not be realized due to the significant time delay between the deposition and freezing. At an architectural scale, ice construction has been proposed as a possible solution for building human habitats on Mars.^[^ ^12^ ^]^ Several groups have also attempted to print structures at freezing temperatures, including liquid metal,^[^ ^13^ ^]^ aerogels,^[^ ^14^ ^]^ and hydrogels.^[^ ^15^ ^]^ Other researchers have proposed using water ice as a positive resist for 3D nanofabrication using ice‐assisted electron‐beam lithography (i.e., ice lithography).^[^ ^16^, ^17^ ^]^ A thin film of ice, deposited and selectively etched inside an electron microscope, is used as a sacrificial mask layer for fabricating 3D nanostructures with complex features including overhanging geometries. By stacking multiple layers, the process can also be used to fabricate multi‐layered structures in fewer steps compared to conventional techniques.^[^ ^18^ ^]^

We present a support‐free, freeform 3D ice‐printing process for the high‐speed fabrication of complex, mesoscale ice structures with micro‐scale resolution and smooth surfaces. The dimension and resolution of the printed ice features are dictated by essentially controlling the droplet deposition frequency (frequency modulation) and the X–Y motions of a high‐precision motion system. Experimental studies are performed to determine the printing path, motion‐stage speed, and droplet frequencies that enable the reproducible fabrication of smooth ice structures with straight, inclined, branching, and hierarchical geometries. Several sample ice structures are printed to demonstrate the proposed technique. Finally, we demonstrate an innovative reverse‐molding application of the freeform 3D ice printing technique by printing sacrificial ice templates to fabricate parts with well‐defined internal features/voids.

## 2 Freeform 3D Printing of Ice

**Figure 1:**
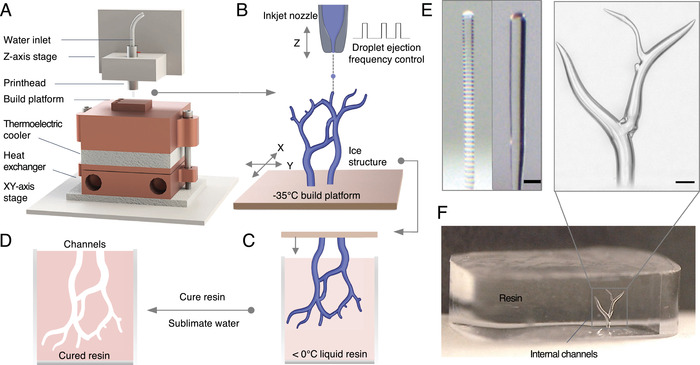
Freeform ice printing (3D‐ICE). A) The custom‐built 3D printing system and its principal components, including the cooling system, motion stages, piezoelectric nozzle. B) A piezoelectric inkjet nozzle is used to eject water droplets (diameter = 50 µm) onto a cold build platform maintained at −35 °C. Planar (X–Y) motion of the build stage is synchronized with droplet discharge to print intricate ice geometries. C) The ice templates can be submerged inside light‐curable, chemically cross‐linkable, or solvent‐based polymers as the external material (solid portion). D) After curing the surrounding matrix, the water is sublimated out to produce the final positive form. E) The low frequency deposition mode produces layered print geometry (left) whereas the high frequency deposition mode produces a continuous, smooth geometry (right). Scale bar is 100 µm. F) Photograph of a branched tree channels in the cured resin (bottom). Our freeform technique enabled by droplet‐ejection frequency modulation synchronized with the stage motions enables printing the tree geometry with smooth surfaces and continuous (non‐discrete) variations in diameter with smooth transitions. The zoomed microscope image (top) demonstrates our ability to accurately capture the microscale features of the ice templates using the reverse molding process. Scale bar is 200 µm in (E,F).

Here, we developed an inkjet‐based freeform 3D freeze printing approach, as depicted in Figure **1**. In this approach, a rapid liquid‐to‐solid phase change was induced to the printable ink (water) through freezing. This rapid phase change and the strength of the frozen ink enabled *freeform* 3D printing of ice structures without requiring support structures. We used 3D‐ICE to fabricate structures with smooth surfaces, continuous (non‐discrete) variations in diameter, and overhanging features. Such structures are very challenging or impossible to fabricate using the voxel‐based methods and are uniquely enabled by our freeform 3D fabrication method with the droplet‐ejection frequency modulation synchronized with stage motions (see Figure S18, Supporting Information, for a demonstration comparing voxel‐based methods with 3D‐ICE). 3D freeze printing was performed on a custom‐built high‐precision 3‐axis motion system. The water droplets were deposited onto a custom‐built temperature‐controlled build platform mounted on an *X*–*Y* stage using an inkjet printhead (Figure 1A,B). The low platform temperature (e.g., −35 °C) freezes the impingent water droplets while maintaining the print volume at temperatures below the freezing point. We used droplet ejection frequency modulation synchronized with stage motions to print 3D ice structures with smooth walls, continuous (non‐discrete) variations in diameter, and overhanging features. The build platform temperature was controlled using a recirculating chiller and a digitally controlled Peltier cooler (Custom Thermoelectric, LLC, Bishopville, MD) (Figure 1A), enabling rapid and precise platform temperature control with 1 °C resolution. The substrate temperature of −35 °C is a current limitation of our hardware. In the future, using lower platform temperatures could enable the fabrication of larger structures at faster printing speeds. The inkjet printhead (MJ‐AB‐01, MicroFab, Inc., Plato, TX) was mounted on a Z‐axis motion stage. The size of the ejected droplets is a function of the nozzle diameter and the voltage waveform applied to the piezoelectric printhead. The motion stages (ALS130‐150, Aerotech, Inc., Pittsburgh, PA) and the inkjet printhead were computer‐controlled and programmed to make precise, sub‐micron resolution movements synchronized with droplet ejection. The system was placed in a custom‐built acrylic enclosure, and a dry nitrogen purge was used to control the humidity inside the print chamber to prevent frost formation on the build platform and constructs during the printing process.

In this work, we introduced and analyzed the 3D‐ICE process using a 50 µm nozzle. The size of the ejected droplets, and therefore the minimum ice feature that can be fabricated, is a function of the nozzle diameter and the voltage waveform applied to the piezoelectric printhead. To fabricate structures with smaller feature sizes, smaller nozzle diameters may be used. To demonstrate this capability, we also created ice pillars with varying diameters using a 20 µm nozzle (Figure S15, Supporting Information). The 20 µm nozzle produced water droplets with a considerably smaller diameter and volume (as the volume decreases with the cube of diameter). This small volume caused the small droplets to freeze much faster than the larger droplets created using the 50 µm nozzle. Therefore, fabricating smooth structures with the 20 µm nozzle required higher droplet‐ejection frequencies. The use of nozzles smaller than 20 µm may bring additional challenges to ice printing, such as in‐flight aerosolization of the water droplets and high sensitivity to contamination, potentially affecting the stability and reliability of the process.

### 2.1 Pillar Diameter Control Using Frequency Modulation

**Figure 2:**
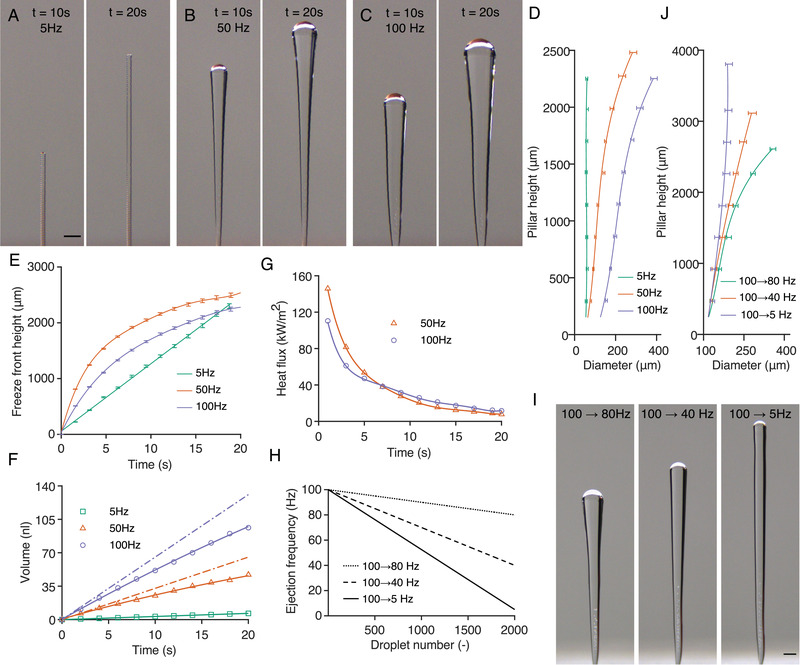
Controlling the geometry of the 3D‐ICE printed pillars using droplet ejection frequency. A– C) Printing droplets at 5, 50, and 100 Hz without moving the stage. Results are shown at times *t* = 10 and 20 s. Higher droplet ejection frequencies result in thicker geometries. Discrete layered deposition is observed at 5 Hz. D) The pillar diameter is plotted against the pillar height for geometries printed at the three frequencies. The pillar diameter increases with increasing deposition frequency. For a constant droplet frequency, the diameter also increases with increasing height due to the reduced heat flux away from the build platform. E) The observed height of the freezing front (measured from the base) as a function of time for the three deposition frequencies. The freeze front location is identified using the minor color difference between the ice and water phases (see Supporting Information). F) The dashed lines indicate total deposited water volume for each deposition frequency as a function of time. The solid lines specify the calculated volume of frozen water in the ice pillars. For direct comparison with deposited volumes, the ice volumes are reported as water equivalents by multiplying them with the water‐to‐ice density ratio. From mass conservation, the difference between the dashed and solid lines indicates the volume of the liquid water cap on top of the ice pillars. G) Heat flux at the freeze front is estimated from the solidification rate of water at the freeze front. The heat flux reduces as the front progresses further away from the base because of the unidirectional cooling in our current setup. Heat flux is also plotted as a function of freeze front height in Figure S2, Supporting Information. H) To print pillars with a constant width, the reduction in heat flux with increased pillar length can be compensated by reducing the droplet ejection frequency. Pillars with three linearly decreasing frequency profiles are printed. I) The printed geometries corresponding to the frequency profiles in (H). For each pillar, 2000 droplets are deposited. J) The change in width with increasing height for the pillars shown in (I). After a short initial conical section, a constant pillar width of 120 µm is observed for the [100 Hz → 5Hz] case. All scale bars are 200 µm. Pillar diameter and freeze front heights in (D), (J) and (E) are reported as ranges, calculated from five repeated experiments. Heat flux and volume are calculated from the mean values of pillar diameter and height.

Controlling the structural dimensions using frequency modulation is an extremely important unique approach with our 3D ice printing. Solving this challenge enabled us to build complex 3D ice structures with high precision and reproducibility, and expanded the capabilities of our 3D ice printing approach tremendously. Controlling the cross‐sectional dimension and geometry of the 3D printed ice structures is critical for fabricating complex geometries. As a prerequisite to printing more complex structures, we first evaluated the fabrication of straight ice pillars with circular cross‐sections without using the X– Y stage motions. The diameter of such posts can be controlled by modulating the droplet ejection frequency. During freeze printing, a liquid pool forms at the print surface (e.g., top of the pillar; Figure S1 and Movie S1, Supporting Information). The cross‐sectional diameter of the liquid pool is inversely proportional to the freezing rate (heat flux) at the freeze front. When the cooling is unidirectional, that is, originated from the bottom platform only, the freezing rate will depend on the length of the pillar since the heat transfer is controlled primarily by conduction. A thicker liquid pool requires a longer duration to freeze. When the droplet ejection occurs below a critical frequency, the individual droplet freezes before the next one arrives. This results in a striated print geometry (Figure 1E, left), where a layered shape is visible. In contrast, the liquid pool consists of multiple droplets at higher frequencies. The liquid pool continuously freezes in the direction of pillar growth, resulting in smooth geometries (Figure 1E, right). The diameter of the liquid pool also increases with deposition frequency, producing printed ice structures with larger diameters (**Figure** **2**A–D)). This relationship enables varying the diameter of the printed ice structures in situ by modulating the droplet ejection frequency.

Figure 2 illustrates the deposition of water droplets onto a stationary stage at different ejection frequencies. The computer control of the piezoelectric nozzle allows us to dictate the time interval between individual droplets accurately. At 5 Hz ejection frequency, individual droplets freeze before the subsequent droplet contacts onto the pillar, resulting in a striated print geometry. In contrast, droplets deposited at 50 and 100 Hz produced pillars with continuous architecture and smooth surfaces (Figure 2B,C; Movies S2 and S3, Supporting Information). Higher deposition frequencies result in ice geometries with larger diameters. The reduced heat flux at longer “cord” lengths (i.e., increased height for the vertical pillars) causes the pillar diameters to increase at a given droplet frequency. (Figure 2D,G)

As such, producing a uniform‐diameter pillar requires compensation of the reduced heat flux by reducing the droplet frequency (increasing the time between subsequent droplets) as the pillar length grows. Modulating the droplet discharge frequency allowed us to continuously vary the width of the printed geometry within a broad size range (≈50–400 µm here), unlike the discrete nature of the layer‐by‐layer or droplet‐wise deposition modes.

To determine the droplet frequency required to print the desired pillar diameter, we considered the dynamics of the freezing process. Conservation of mass dictates that the sum of the ice and liquid water cap masses is equal to the total mass of the deposited water. For the layered deposition at 5 Hz, the mass of the ice structure is equal to the total deposited water mass (up to a certain height) since each droplet freezes before the next one arrives. At higher deposition frequencies, we calculated the volume of the liquid pool from the difference between the total deposited mass and the mass of the ice (see, Supporting Information for details). Note that reduced heat flux at longer pillar lengths affects the droplet frequency needed to produce a required width. For instance, at sufficiently long pillar heights, 5 Hz frequency also produces continuous structures with smooth surfaces (Figure 2I). We calculated the heat flux at the freeze front using the instantaneous freezing rate and freeze front diameter and observed it decrease with time as the front propagated along the cord length due to the thermal gradient (Figure 2G). The volume of the liquid pool directly correlates with the resulting ice pillar diameters post‐freezing. To achieve a desired constant pillar diameter, the droplet ejection frequency was modulated to keep the liquid pool volume constant, accounting for the reduction in heat flux at the freeze front with increasing height. As an example, linearly reducing droplet ejection frequency from 100 to 5 Hz results in a pillar with a constant 170 µm diameter (Figure 2H–J).

The repeatability of the 3D‐ICE approach is critical to its utility as a broadly applicable fabrication process. To this end, we performed a repeatability/reproducibility analysis on fabricated ice pillars. First, we fabricated 25 pillars with 100 µm target diameter size and measured their width from the images. The details of this study are included in Figure S16, Supporting Information. Note that 25 pillars were fabricated in batches of five; thus, the statistics include both repeatability and reproducibility of the process. We note that our measurement approach may have 1–2 µm uncertainty arising from the wavelength of the visible light and the lighting conditions/light reflections from the ice pillars. We created a histogram of the data and conducted a normality test. The results indicated that the variation in diameter followed a normal distribution, with an average width (diameter) of 102 µm and a standard deviation of 4 µm. Second, we conducted five repetitions for each pillar used for the study (Figure 2) and added error bars (range) to Figure 2D,J,E.

### 2.2 Shape Control Using X–Y Stage Motions

In addition to the droplet ejection frequency, the print geometry is dictated primarily by the X–Y stage motions during printing. Using controlled stage motions coordinated with the droplet dispensing allowed us to print ice forms with complex geometries, such as steep overhang angles or arch‐type structures. Precise synchronization of stage motions and droplet dispensing is critical to enable free‐form 3D printing without sacrificial support structures.

**Figure 3:**
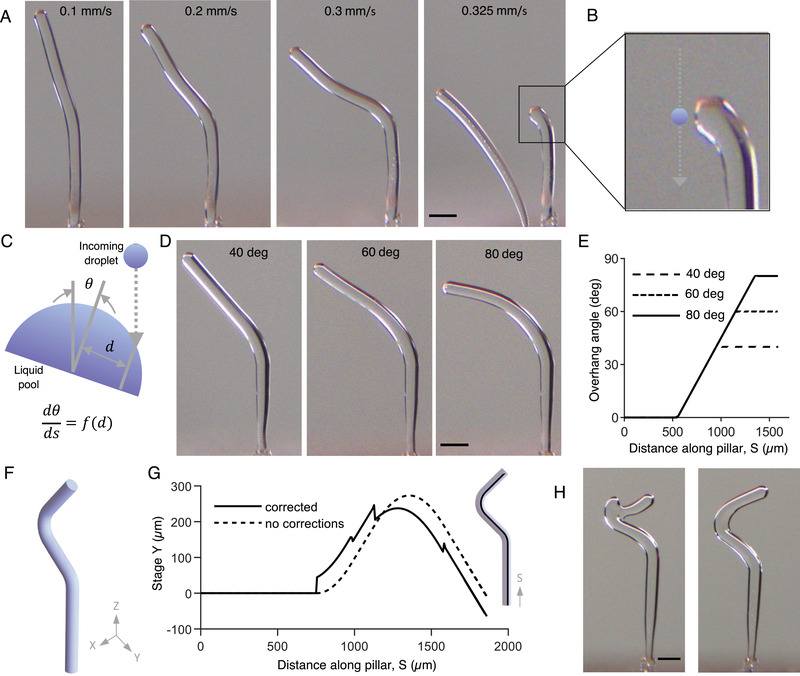
Support‐free overhanging ice forms through control of stage motion coordinated with droplet deposition. A) Pillars printed by moving the *Y* stage at constant velocities of 0, 0.1, 0.2, and 0.3 mm s^−1^. Faster stage motion results in steeper overhang angles. Eventually, at a *Y*‐stage velocity of 0.325 mm s^−1^, the motion is too fast for the freezing rate, and the incoming droplets do not impinge on the existing pillar surface and start to form a new pillar (B). Each pillar was printed in ≈3.5 s. The ratio between the stage translation and freeze‐front growth speed determines the heading angle (angle between the pillar growth direction and vertical) of the freeze front. The faster that the stage moves relative to the freeze‐front growth, the steeper is the heading angle. The pillars in (A) get progressively steeper as the ratio between the stage and freeze front propagation speeds increases with time. C) Moving the stage between sequential droplets results in the off‐axis deposition of the incoming droplet. This asymmetry changes the direction of the freeze front toward the direction of the incoming droplet with a rotation rate dependent on the droplet offset distance. Ratios of stage translation velocity to freeze front growth speed are reported in Figure S14, Supporting Information. D) Pillars printed at constant inclination angles of 40°, 60°, and 80°. E) The overhang angle (measured from the vertical) along the length of the pillars demonstrating our ability to use freeform ice printing with desired tilt angles. F) A CAD model for a simple zigzag structure. G) The stage motion trajectories for printing the 3D model illustrated in (F). Simply moving the stage along the center‐line of the desired geometry (dashed line) results in an inaccurate print geometry (H left). When the corrections are applied to the print path based on the curvature of the geometry (solid line), we are able to print the desired form (H right). All scale bars are 200 µm.

**Figure** **3** demonstrates the fabrication of angled and curved ice structures using stage motions coordinated with droplet ejection. The local slope of the resulting structures depends primarily on the ratio of the stage speed and the rate of growth of the freezing front. For a given droplet frequency, increasing the speed of the X–Y stages produced geometries with steeper angles. When the X–Y stage moved between sequential droplets, the resulting off‐axis deposition of the incoming droplets produced a local inclination. This resulted in a gradual rotation of the freeze front in the deposition direction. However, excessive stage displacements between subsequent droplet ejections (as caused by large stage velocities) resulted in the approaching droplet to miss the liquid cap and deposit onto the platform, starting a new pillar (Figure 3B).

At a given pillar length, the rate of the freeze front rotation depends on the offset of the incoming droplet from the heading axis at the pillar tip (Figure 3C). We used this information to generate stage motion trajectories to print inclined and curved geometries. This approach formed the basis for printing more complex shapes. To print a given curved shape, the print path is generated by adding a correction factor to the centerline of the geometry. For example, consider the simple zigzag structure in Figure 3F. Simply moving the stage along the centerline (Figure 3G dashed line) results in an inaccurate print geometry (Figure 3H, left). To compute the correction factor, we first calculate the rate of change of the slope along the length of each branch of the print geometry. The correction factor is then calculated as an offset from the centerline using the inverse of the experimentally determined function from Figure 3C. Using this correction based on the curvature of the geometry (Figure 3G, solid line) on the print path enabled the successful fabrication of the desired form (Figure 3H, right). At this size scale, gravitational forces do not play a significant role, and surface tension allows the liquid droplet cap to tightly adhere to the ice pillar even at very steep angles (see the bond number calculation in Supporting Information). Using our freeform printing technique, we demonstrated the printing of inclined pillars with overhangs as steep as 80° (measured from the vertical) (Figure 3D,E and Movies S4– S6, Supporting Information, corresponding droplet ejection frequencies and stage motions in Figures S3– S5, Supporting Information). Although we present the results for the *Y*‐axis motions, the results are equally applicable for any stage motions in the X–Y plane. Thus, the resulting motion can be used to generate complex ice geometries with 3D features.

### 2.3 Printing Complex 3D Ice Structures

**Figure 4:**
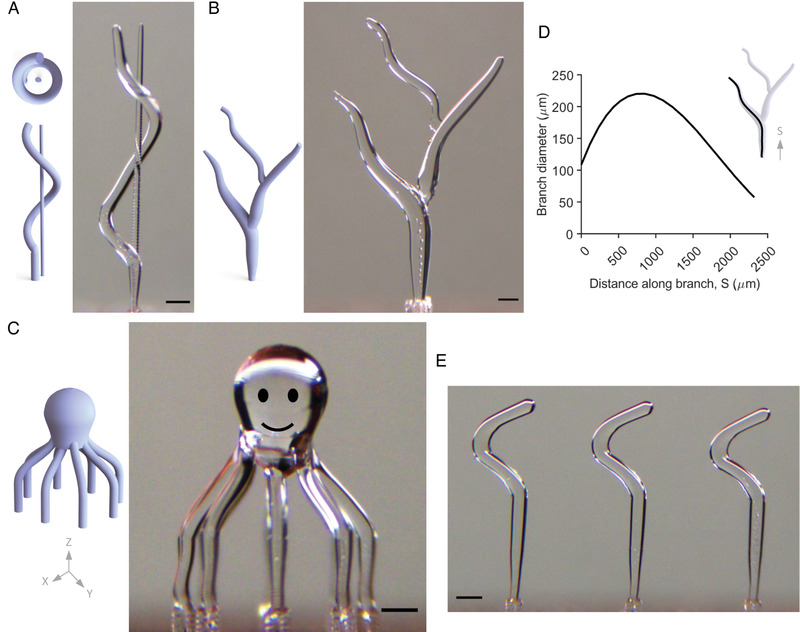
3D‐ICE printing of complex 3D geometries with micro‐scale features and smooth walls. A) Helical coil 3D geometry with an independent central pillar showing the ability to print intertwined structures. The helix diameter is 400 µm, the strand diameter 100 µm, and the pillar diameter is 50 µm. B) A branched tree structure with varying branch diameters. C) An octopus geometry with eight 90 µm diameter tentacles shows the ability to integrate small elements into complex forms. The individual tentacles (with steep overhang geometries) are printed first. The body/head portion was printed subsequently. The three models were printed in 43, 38, and 57 s, respectively. The eyes and mouth are added in the octopus geometry using image processing for artistic effect. D) Measured width along the main branch of the tree showing our ability to smoothly transition between increasing and decreasing pillar sizes by varying the droplet ejection frequency. E) Three repeats of the zigzag geometry from Figure 3F, demonstrating our ability to print repeatably. All scale bars are 200 µm.

To demonstrate the capability of our freeform ice printing technique, we fabricated three designs to demonstrate the key features of smooth cylindrical surfaces, sections with varying diameters and branched structures with smooth transitions between the branches. **Figure** **4** shows the three different 3D models: a helical coil with an independent central pillar, a branched tree‐like structure, and a microscale octopus sculpture. Based on the CAD model of the geometry, we generated the print code that specified the time‐dependent droplet ejection frequencies and the associated *X*– *Y* stage motions. In most cases, the stage remained stationary during droplet ejection, and stage motions occurred in the time interval between ejection of individual drops. For these prints, the nozzle *Z*‐height was held constant. For the helical coil, the helix structure was first printed in one continuous step, followed by the thinner central pillar (Figure 4A; Movie S7, Supporting Information). For the branching structure, the main branch (trunk) anchoring the tree to the build surface was printed first, followed by the thicker branch and the thinnest branch. We varied the branch thickness along the length by systematically varying droplet deposition frequency (Figures 4B,D; Movie S8, Supporting Information). Essentially, at any given time instant, if the deposition rate exceeded the freezing rate, the diameter of the ice pillar started to increase. To obtain a smooth transition from the trunk to a branch, we deposit multiple droplets side by side. The tree‐like geometry demonstrates our ability to print a hierarchically branched structure with smooth surfaces and transitions, and continuously varying diameters. The octopus ice sculpture was printed in nine steps, with the eight legs printed first. After printing all eight legs (one at a time with varied orientations), the head/main body was fabricated by depositing droplets when the stage was moving (Movie S9, Supporting Information). The droplet deposition frequencies for the prints ranged from 5 to 300Hz, and each complex structure took less than a minute to print, underscoring the rapid nature of the freeform printing process. Additional complex shapes printed using out 3D‐ICE process are shown in Figures S6 and S7, Supporting Information.

### 2.4 Reverse Molding of the Printed 3D Ice Templates

An exciting and compelling application of 3D ice printing is its use for fabricating sacrificial templates. Such ice templates can be used for reverse molding, that is, to create internal (negative) channels and other void features in a solid (positive) part.^[^ ^19^ ^]^ The ability to sublimate out the ice in a lyophilizer allows for its easy removal after casting and solidification of the surrounding positive form (e.g., resin). Alternatively, when the process is used to make channels in hard materials, the ice can be melted into water and easily removed via expulsion or evaporation. However, using a similar process for soft and delicate positive forms can damage these materials and collapse the fabricated channels due to surface tension forces. In this case, our approach to directly sublimate away the sacrificial (ice) templates causes minimal disruption to the positive form. Potential applications of reverse molding include, but are not limited to, soft matter and electronics manufacturing,^[^ ^20^, ^21^ ^]^ biomedical device fabrication,^[^ ^19^, ^22^ ^]^ tissue engineering,^[^ ^23^, ^24^, ^25^ ^]^ and even art. Previously, ice has been used as a sacrificial support material for fused deposition modeling^[^ ^26^ ^]^ and stereolithography.^[^ ^27^ ^]^ Alternatively, cryogenic printing has also been used in tissue engineering for printing layered scaffolds with aqueous polymer slurries.^[^ ^28^, ^29^, ^30^ ^]^ After printing, these constructs were freeze‐dried to sublimate the water, leaving behind a porous scaffold. However, layered deposition during the printing process results in a striated scaffold structure, which is generally undesirable. Furthermore, the existing approaches only demonstrated simple, grid‐based geometries. Recently, sacrificial ice printed templates dip‐coated in extracellular matrix proteins have been used for fabricating personalized tissue grafts with hierarchical vascular branches.^[^ ^31^ ^]^ However, this method could only be applied to produce large freestanding ice structures with minimum dimensions greater than several millimeters.

**Figure 5:**
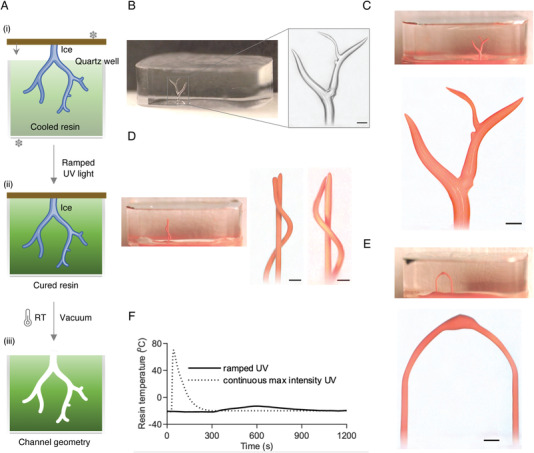
Reverse molding channel geometries by casting a UV‐curable resin around the 3D ice templates. A) Schematic illustration of the reverse molding process. The printed ice templates are submerged into pre‐chilled photo‐curable resin. A digitally‐controlled UV lamp cures the resin around the immersed ice forms. Upon warming, the ice liquefies to water and is evacuated using vacuum, leaving behind the corresponding channel geometry. B) Photograph of branched tree channels in the cured resin (left). The zoomed microscope image (right) demonstrate our ability to accurately capture the microscale features of the ice templates using the reverse molding process. C– E) Three examples of shaped void geometries created in resin– the branched tree structure, a helix with an independent central column and an arch. The channels are perfused with an opaque orange stain for improved visualization. The helix is imaged in two different orientations to show its 3D structure. Inset images are digital photographs of the corresponding stain‐filled geometries. F) Measured resin temperatures during the ultraviolet‐curing process. Active cooling and ramped irradiation intensity over 20 min prevented the resin temperature from exceeding the melting point of ice. After an initial spike at the start of the curing reaction, the temperature slowly drops down even as the intensity of the UV light is increased. In contrast, continuous high intensity exposure rapidly cures the resin resulting in a huge temperature spike. All scale bars are 200 µm.

Here, we demonstrate the rapid fabrication of parts with micro‐scale internal geometries from a light (ultraviolet, UV)‐curable resin. As described in **Figure** **5**A, the resin was first cooled down to −15 °C to prevent the melting of the ice templates; the resin remained liquid at this temperature. Next, the 3D‐printed ice structure was submerged into the liquid resin using our custom‐built automated apparatus (Figure S8, Supporting Information). After immersing the ice into the resin, we used a variable intensity UV‐lamp (Figure S9, Supporting Information) to slowly cure the resin in 20 min. The ultraviolet‐curing reaction used for curing the encapsulating resin is exothermic. However, curing kinetics studies in the literature have shown that the reaction rate and resultant heat release rates are proportional to the irradiant light intensity.^[^ ^32^ ^]^ Therefore, to prevent melting the submerged ice structures, we actively cooled the resin and slowly ramped the curing UV lamp intensity over 20 min. This maintained the temperature of the encapsulant well below the melting temperature of ice (Figure 5F). Figures S10 and S11, Supporting Information, illustrate our initial attempts at curing the resin with a continuous high‐intensity UV‐light. The resulting high temperatures melted and displaced the ice templates, resulting in a tremendous mismatch between the indented and actual channel geometry, underscoring the importance of our approach of controlling UV‐light intensity during the curing process. After curing the resin, the water was removed by evaporation, expulsion, or sublimation, leaving behind the final positive geometry with well‐defined internal features and conduits that replicated the printed ice geometries. In this work, we used an optically transparent resin to enable imaging of the fabricated parts using optical microscopy. To better illustrate the porosity of the parts, we filled the conduits with an orange‐colored opaque dye (Microfil MV‐117, FlowTech Inc., Boulder, CO) and imaged the parts using a digital‐optical microscope (Figure 5C–E).

We performed a repeatability study for the inverse molding process. Five ice pillars with nominally the same diameter (100 µm) and spacing (500 µm) were fabricated and used to create internal channels in UV‐curable resin. The average internal channel diameter was measured as 98 µm with a standard deviation of 3 µm. The spacing between the channels was measured as 507 µm with a standard deviation of 5 µm (Figure S16, Supporting Information).

## 3 Conclusion

This work introduced the freeform 3D ice printing (3D‐ICE) process to fabricate ice structures with micro‐scale resolution. Our freeform printing strategy eliminates the need for support structures and layer‐by‐layer construction, enabling high‐speed and reproducible fabrication of complex 3D ice structures with micro‐scale resolution, continuously varying circular cross‐sections, smooth surfaces, and branched hierarchical geometries with smooth intersections. The dimension and resolution of the printed ice features were dictated by modulating the droplet deposition frequency and the X–Y motions of a high‐precision motion system. Experimental studies were performed to determine the printing path, motion‐stage speed, and droplet frequencies to reproducibly fabricate smooth ice structures with straight, inclined, branching, and hierarchical geometries. Notably, the knowledge of freeze‐front progression allowed continuous and rapid variation of the printed feature size even when the X–Y stages were stationary. We printed several sample 3D ice structures to demonstrate the proposed technique.

An innovative application of the freeform 3D ice printing technique was realized by printing sacrificial ice templates to fabricate parts with well‐defined internal features/voids. The printed 3D ice templates were submerged into a UV curable resin. The water was then sublimated or flowed out to create the final solid components with internal features. This ability to rapidly and easily remove the sacrificial structure from ice to liquid or directly to vapor provides a great advantage, for example, for soft elastomer parts for microfluidics. For parts with medium‐to‐low volume of internal features/voids, this freeform inside‐out 3D printing approach (reverse molding) is orders of magnitude faster than the traditional, layer‐by‐layer positive 3D printing approaches and enables considerable time and cost savings compared to 3D printing of the positive forms. Such components are commonly encountered in 3D microfluidic devices, complex mechanical systems with embedded cooling channels, vascularized engineered tissue scaffolds, and pneumatic actuators for soft robotics. Furthermore, our freeform approach eliminates stepping patterns on surfaces, creating high‐quality parts without time‐consuming and costly finishing processes. The use of water–an abundant, biocompatible, and environmentally friendly resource–as the negative ink makes this technology especially attractive for biomedical applications, 3D microfluidic device fabrication, manufacturing biocompatible soft and flexible electronics, and even for creating artistic structures.

## 4 Experimental Section

### Freeze Printing

Inkjet ice printing was accomplished by modifying the previously described custom inkjet‐based printer.^[^ ^33^, ^34^, ^35^, ^36^, ^37^ ^]^ A piezoelectric droplet‐on‐demand printhead with a 50 µm nozzle (Microfab Technologies, Inc., Plano, TX, USA) was used to produce the water microdroplets for printing. Inkjetting was performed using a sinusoidal waveform with a period of 10 µs and amplitude of 17.0 V, resulting in 50 µm diameter droplets. The nozzle was mounted onto an *Z*‐axis stage (Aerotech Inc., Pittsburgh, PA, USA; Figure S12, Supporting Information). During printing, a standoff distance of 5 mm was specified from the nozzle to the stage. In this study, the nozzle was not moved in the *Z*‐direction during printing. Deionized purified water was used as the ink to ensure consistent freezing behavior. The water used for printing was in a glass vial and fed to the inkjet nozzle via polytetrafluoroethylene (PTFE) tubing. A custom‐built manually‐controlled pneumatic system was used to adjust back pressure at the printhead to achieve stable ejection of water droplets. Additionally, the pneumatic system was also used to apply positive pressure to the water to purge it from the nozzle for cleaning the nozzle prior to printing.

Printing was done on a 12 × 15 mm copper print plate. The print plate was attached to the cooled print platform using a vacuum chuck to prevent unintended relative lateral motion between the motion stage and build surface. The platform was cooled using a two‐stage system consisting of a Peltier cooler (25412‐5L31‐07CQQ Custom Thermoelectric, LLC, Bishopville, MD, USA) sandwiched between a heat exchanger (WBA‐1.62‐0.55‐CU‐01 Custom Thermoelectric, LLC) and the copper vacuum chuck. Indium foil (Custom Thermoelectric, LLC) was used to ensure good thermal contact between the layers. A recirculating chiller (Vevor USA) was used to cool HC‐50 coolant (Dynalene, Inc., Whitehall, PA, USA) to −20 °C, which was circulated through the heat exchanger for the first stage of cooling. The Peltier cooler provided the next stage of cooling. A thermal controller (ETC‐AED330A‐24 Custom Thermoelectric, LLC) was used to switch the Peltier cooler on and off in response to platform temperature measured using a thermistor (Mouser Electronics, Mansfield, TX, USA), allowing to control the temperature of the build stage to within +/−1 °C.

The cooling assembly was mounted onto an X–Y stage (ALS130‐150, Aerotech Inc., Pittsburgh, PA, USA) through a thermally insulating Teflon block. The XYZ stages and droplet dispenser were controlled using a single A3200 Npaq motion controller (Aerotech Inc., Pittsburgh, PA, USA). To eject a single droplet, the motion controller sent a high‐speed digital pulse to the JetDrive controller (Microfab Technologies, Inc., Plano, TX, USA), which applied the analog jetting waveform to the piezoelectric nozzle to eject a droplet.

To prevent frost formation on the cold build plate, the entire setup was contained inside a custom‐built acrylic enclosure. Before starting the printing, the chamber was purged with dry nitrogen for at least 15 min to eliminate ambient humidity. The nitrogen inlet was specifically designed to prevent the formation of recirculation zones and allow uniform dispersion of dry nitrogen gas throughout the chamber (Figure S13, Supporting Information). The nitrogen was turned off during the actual printing process to minimize air currents around the build surface.

Print shapes were designed in SolidWorks (Dassault Systemes SolidWorks Corporation, Waltham, MA, USA) computer aided design (CAD) software and then transferred into MATLAB (MathWorks, Portola Valley, CA, USA). Spline functions were fit to individual print segments. Print stage motions were then generated from segment centerlines, and a correction factor was calculated based on the local segment curvatures. Droplet ejection frequencies were calculated based on desired local segment width and height above the build surface (to account for the vertical thermal gradient in this setup). Final print paths were compiled in the AeroBasic language (Aerotech Inc., Pittsburgh, PA, USA) and were uploaded to the controller to print the desired shapes. Running the code in real‐time on the motion controller allowed coordination between the stage motion and high‐speed droplet ejection.

The printing process was observed using a color camera (MU1203‐BI, Amscope) coupled to a long working distance objective (Infinity Photo‐Optical Company, Centennial, CO, USA), resulting in a 3.7 × 4.9 mm field of view. A bright white laser‐emitted diode (LED) bulb was used to illuminate the scene, and a gray back‐drop was used for imaging. An LED strobe synchronized to droplet ejection was used to view the jetted droplets and ensure consistent jetting of water droplets before the start of the printing. Acquired images were processed using Fiji.^[^ ^38^ ^]^

### Fabrication of Resin Parts with Ice‐Template‐Based Internal Porosity

The printed ice templates were submerged into a UV‐curable resin (Henkel Loctite 3971 Ellsworth Adhesives Germantown, WI, USA) using a custom‐built automated mechanism (Figure S8, Supporting Information). The motion was achieved using the same three‐axis stages used for the printing process. The copper print plate had cylindrical neodymium magnets attached to it for the arm to grab, lift, and flip the plate (more details and figures in Supporting Information). To release the plate from the stage and allow the arm to lift it, the vacuum from the chuck was released before the lifting step. The resin was pre‐chilled using a second Peltier device mounted on the cooled copper stage to prevent melting of the templates before curing, which was critical due to the rapid change in temperature that could occur.

The resin was contained inside a custom‐fabricated well with quartz walls (Technical Glass Products, Inc. (TGP), Painesville Twp., OH, USA) and a thin aluminum base. The high level of transparency of the quartz glass at the UV wavelength facilitated efficient curing of the contained resin and clear optical imaging post‐fabrication. The encapsulation process was observed with a microscope camera (Dunwell Tech Dino‐Lite, Torrance, CA, USA) with a wide field of view. The resin was cured using a high power 400–410 nm ultraviolet LED (LED Supply, Randolph, VT, USA) controlled by an LED driver (LED supply; Figure S9, Supporting Information). The UV light intensity was ramped up slowly to prevent excessive exotherms inside the resin (Supporting Information). After curing the encapsulating resin, the constructs were brought up to room temperature to melt the encased ice. The liquid water was evacuated from the channels using vacuum. The resulting void space was then filled with an orange‐colored opaque stain (Microfil MV‐117, FlowTech Inc., Boulder, CO) using a vacuum filling process. ^[^ ^39^ ^]^ The resulting constructs were imaged using a digital microscope (VHX‐5000, Keyence America, USA) under a combination of ring and coaxial illumination.

### Statistical Analysis

The sample size (*n*) for statistical analysis was chosen as *n* = 5. In one case, 25 repeated experiments were conducted to better understand the nature of statistical distribution of data. MATLAB (MathWorks, Portola Valley, CA, USA) was used to calculate the mean and standard deviation (SD). Error bars were reported as measured range. Repeatability of the printing and reverse molding process are further characterized in Figure S16, Supporting Information.

## Conflict of Interest

The authors declare no conflict of interest.

## Supporting information

Supporting Information

Supplemental Movie 1

Supplemental Movie 2

Supplemental Movie 3

Supplemental Movie 4

Supplemental Movie 5

Supplemental Movie 6

Supplemental Movie 7

Supplemental Movie 8

Supplemental Movie 9

## Data Availability

The data that support the findings of this study are available from the corresponding author upon reasonable request.
