# Supplementary material for: Freeform 3D Ice Printing (3D‐ICE) at the Micro Scale
Source: Adv Sci (Weinh). 2022 Jul 6;9(27):2201566. doi: 10.1002/advs.202201566 (PMC9507341; doi:10.1002/advs.202201566)
Supplement: Supplementary file 1 — Supporting Information [file ADVS-9-2201566-s003.pdf]

ADVANCED SCIENCE
Open Access

Supporting Information

for *Adv. Sci.*, DOI 10.1002/advs.202201566

Freeform 3D Ice Printing (3D-ICE) at the Micro Scale

Akash Garg, Saigopalakrishna S. Yerneni, Phil Campbell, Philip R. LeDuc and O. Burak Ozdoganlar**

Supporting Information for Freeform 3D Ice Printing (3D-ICE) at the Micro Scale

Akash Garg Saigopalakrishna S. Yerneni Phil Campbell Philip R. LeDuc O. Burak Ozdoganlar**

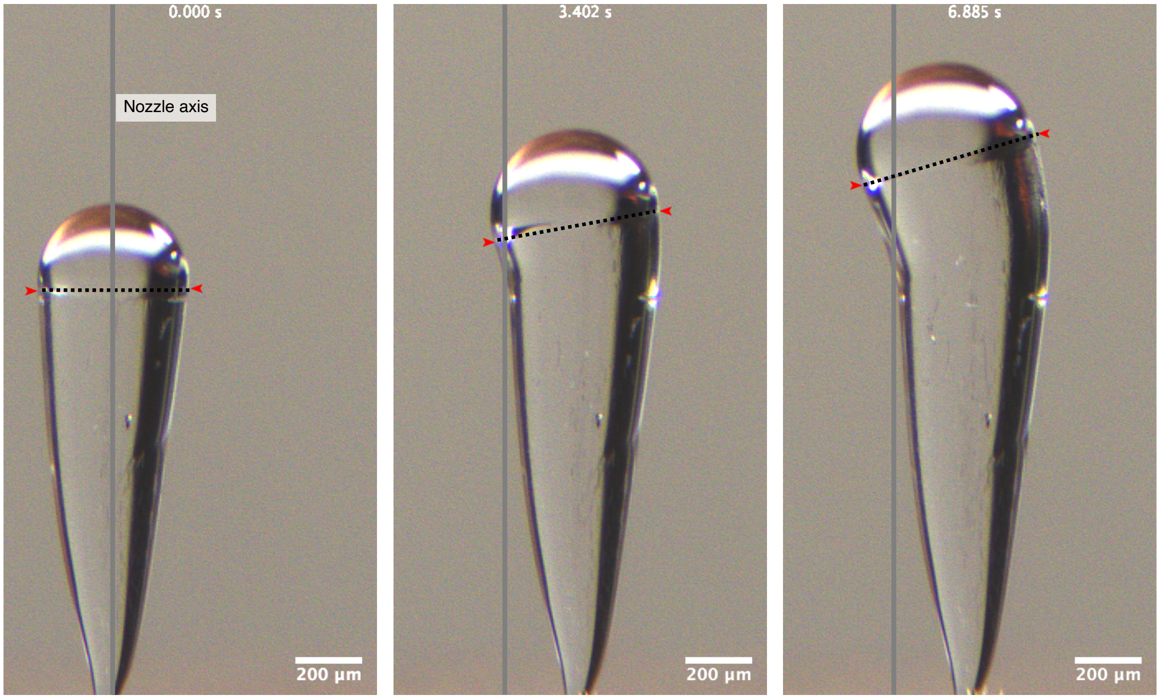

Figure S1: **Three frames from Video S1 illustrating a turning freeze front.** In each image the dashed black line indicates the freeze front, identified as the interface between the transparent water and the slightly translucent ice. The solid gray line shows the nozzle axis. The hemispherical cap above the freezing front is the liquid water cap.

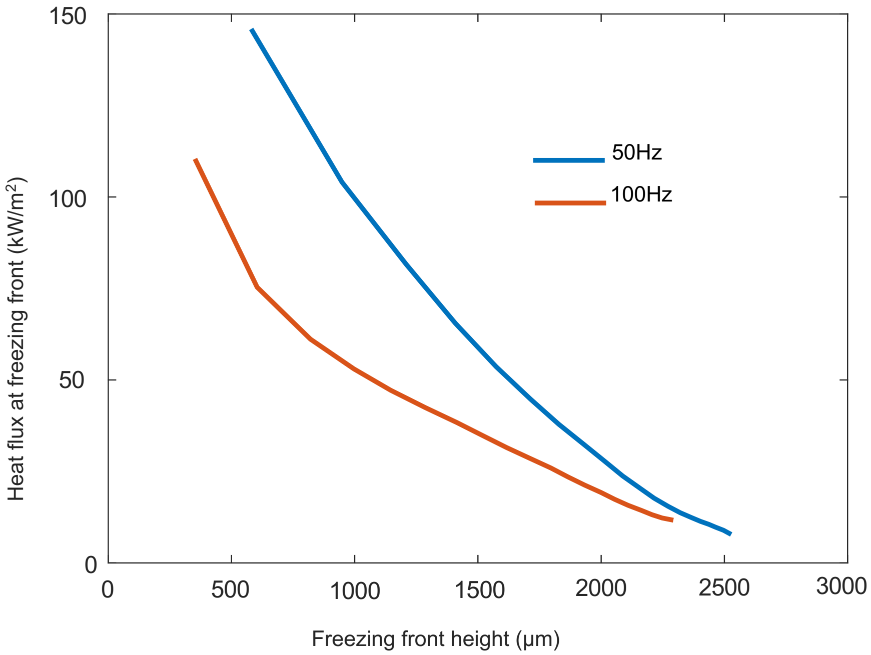

Figure S2: Heat flux at the freezing interface plotted against height of freezing front for constant frequency droplet deposition at 50 and 100Hz.

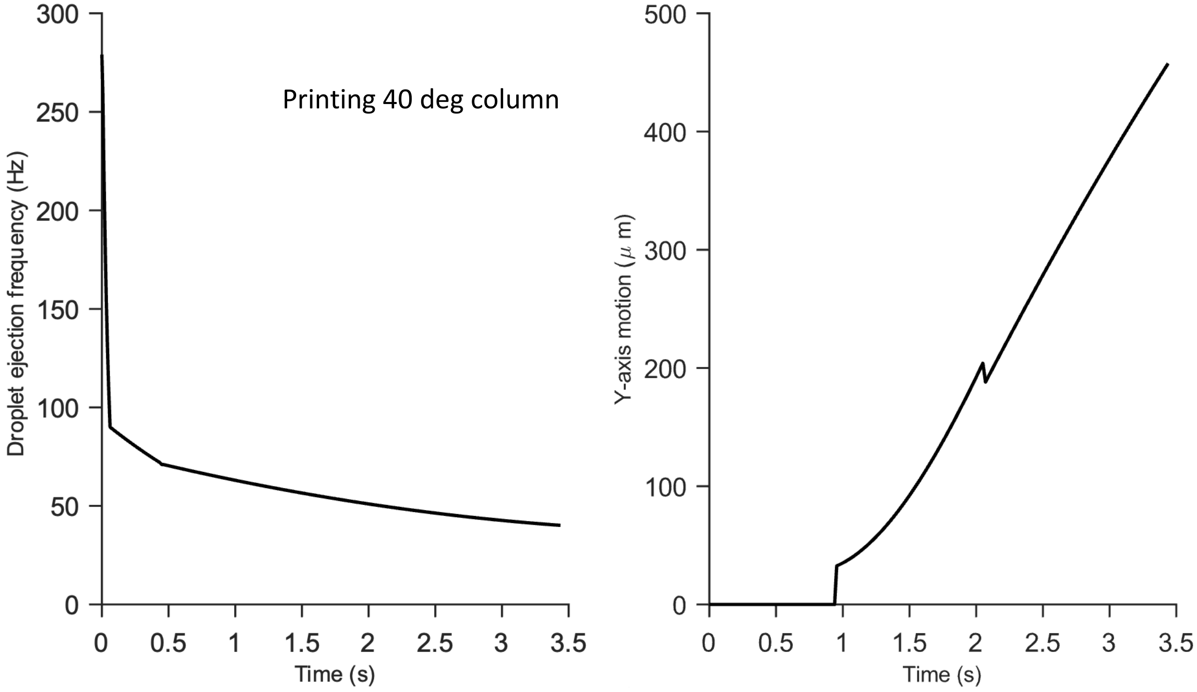

Figure S3: Time history of droplet ejection frequency and stage Y-axis motion for printing a column inclined at 40 deg (see Figure 3(D)).

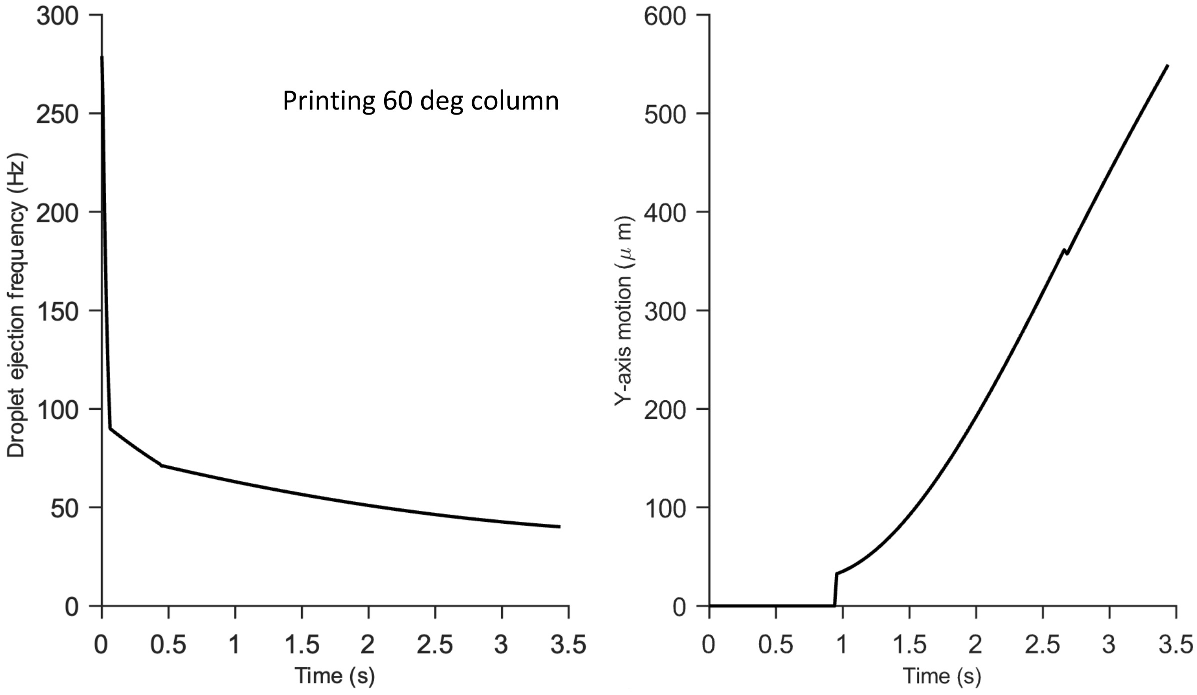

Figure S4: Time history of droplet ejection frequency and stage Y-axis motion for printing a column inclined at 60 deg (see Figure 3(D)).

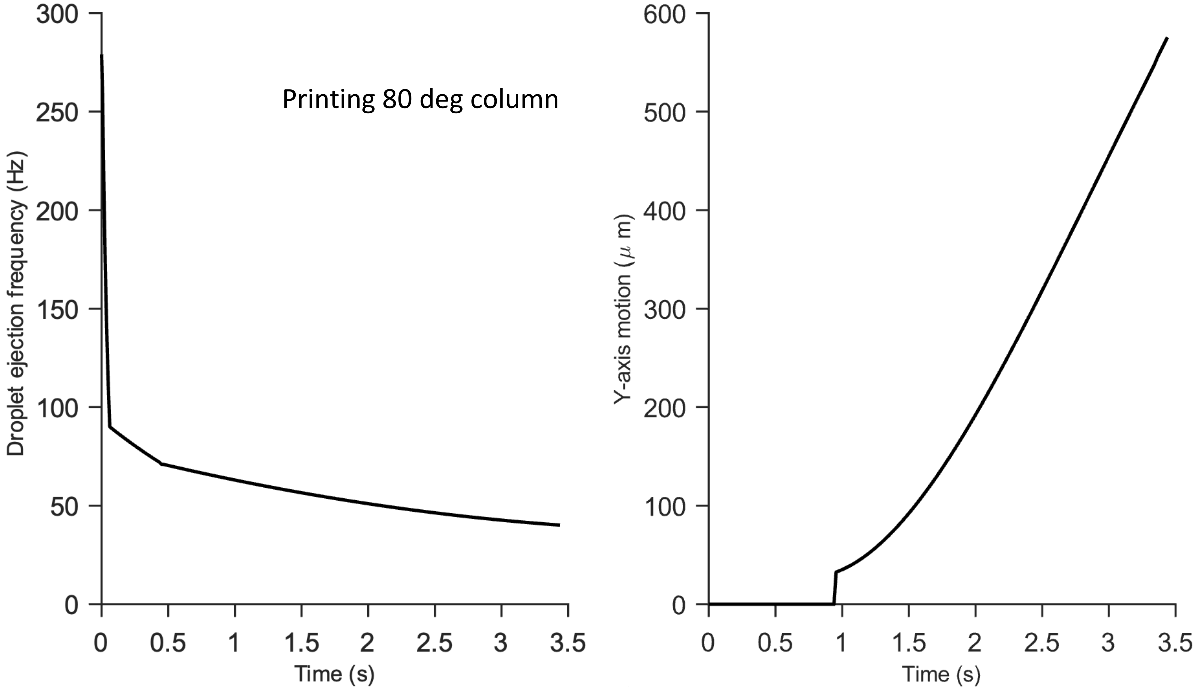

Figure S5: Time history of droplet ejection frequency and stage Y-axis motion for printing a column inclined at 80 deg (see Figure 3(D)).

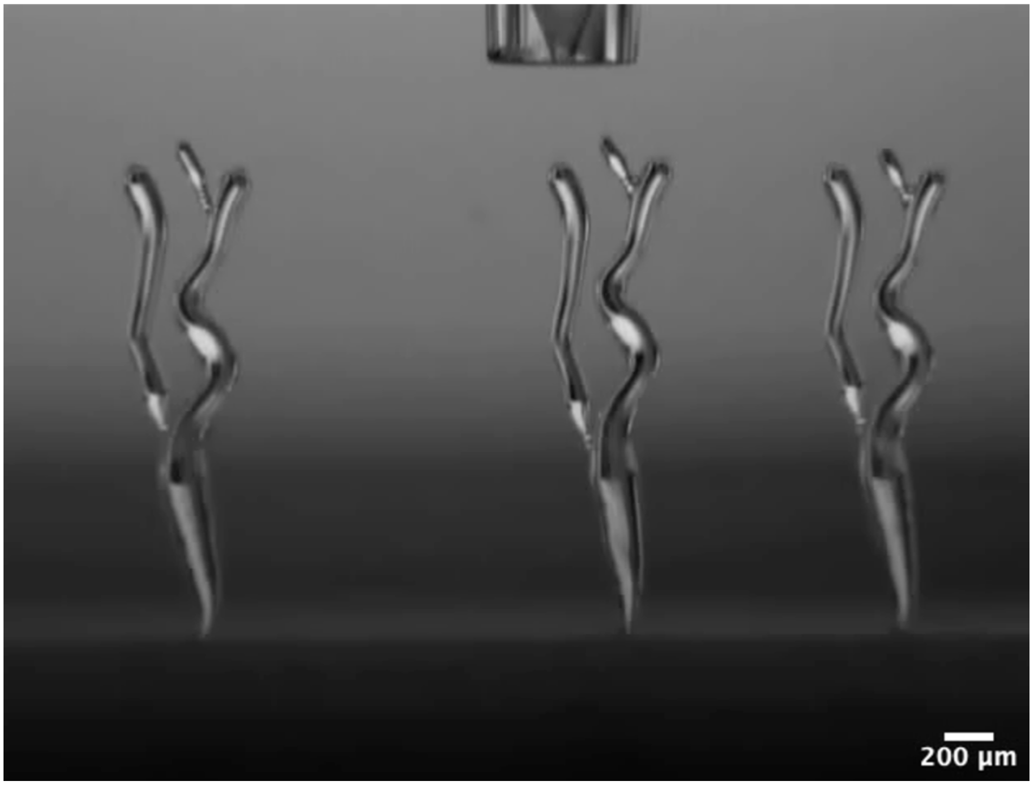

Figure S6: Three repeats of another branched tree structure printed using 3D-ICE.

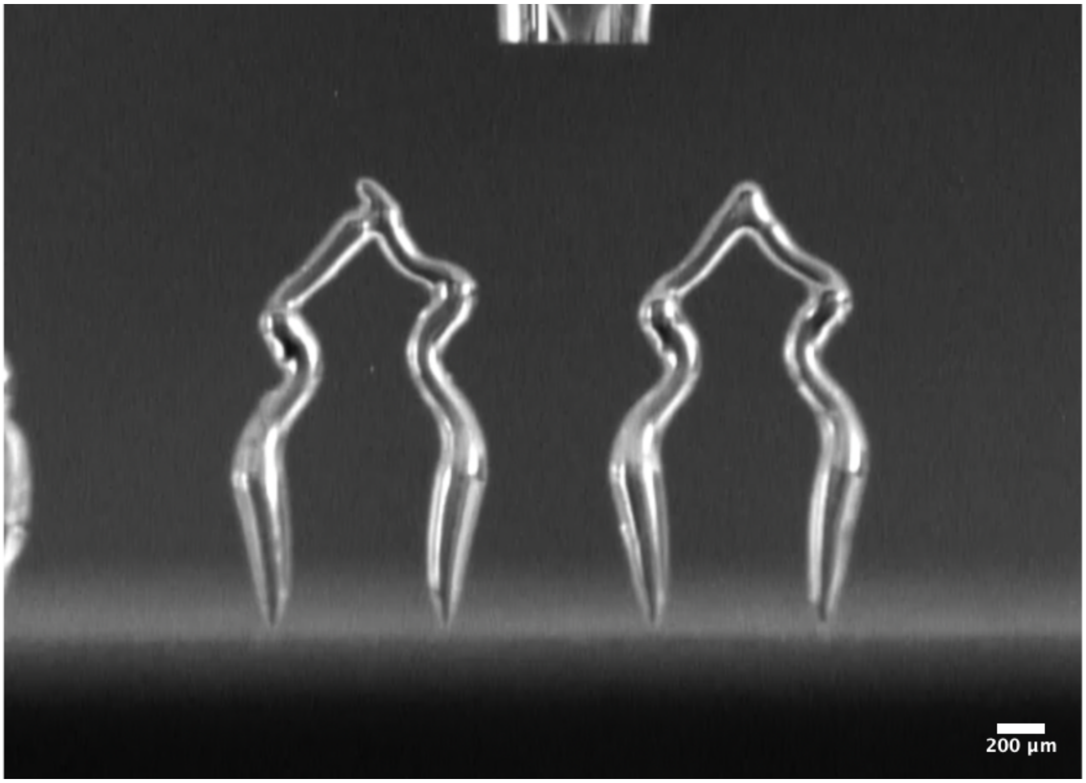

Figure S7: Two repeats of a loop structure printed using 3D-ICE.

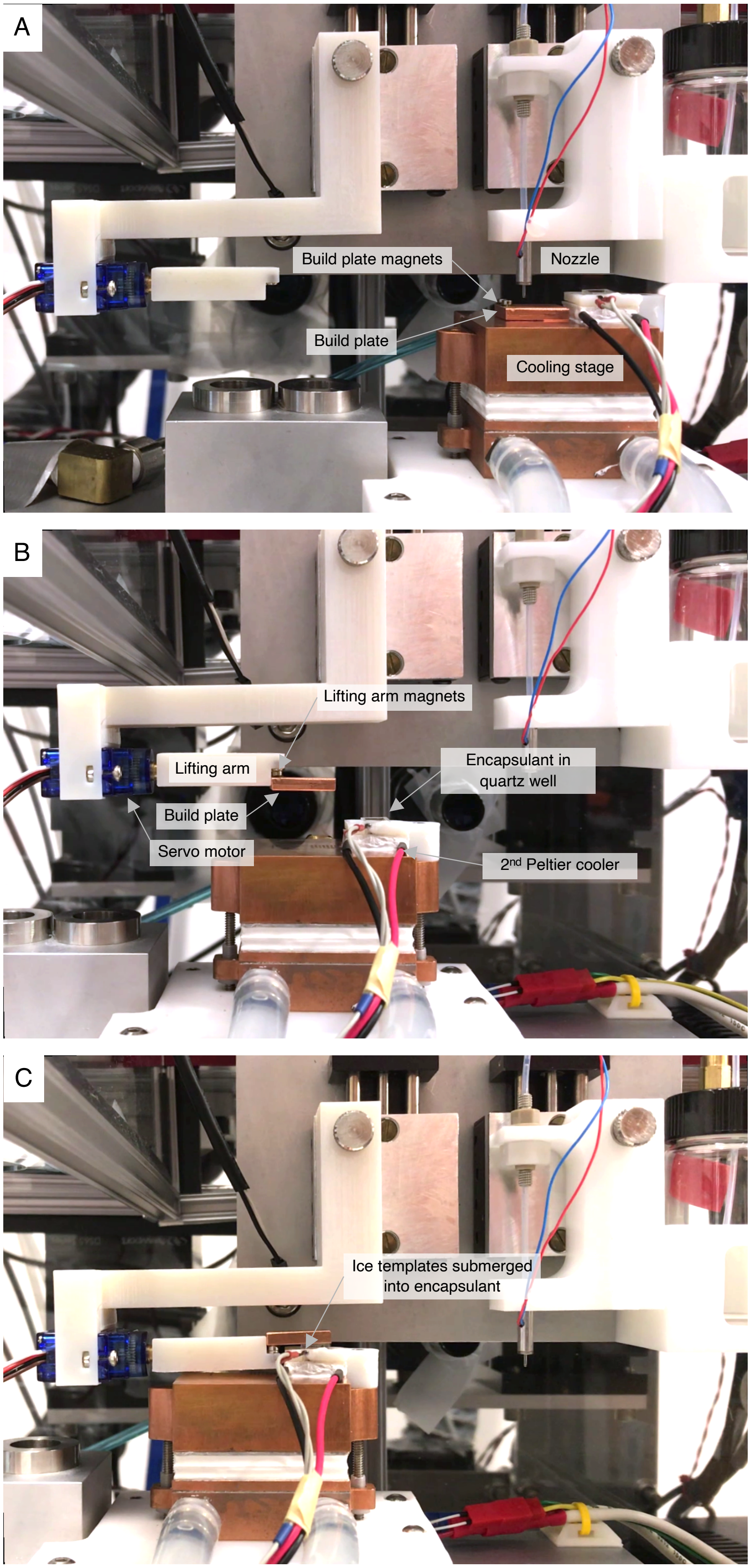

Figure S8: **Custom-built apparatus for submerging ice templates into resin.** **(A)** Ice geometries are printed on top of the build plate. The copper build plate has two cylindrical neodymium magnets glued onto it. **(B)** After the printing is complete, the build plate is moved underneath the lifting arm. The lifting arm is attached to the Z-axis stage, allowing it to move in the vertical direction. A servo motor on the lifting arm can rotate the attached build plate. **(C)** The build plate is flipped and slowly lowered into the resin until the ice templates are completely submerged.

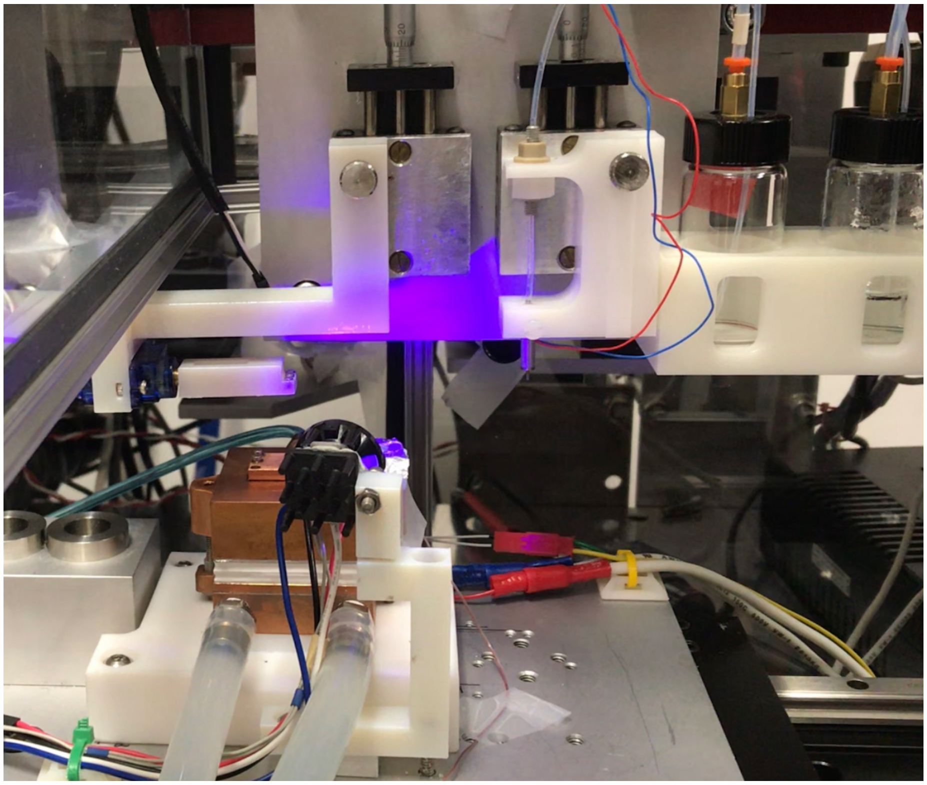

Figure S9: Variable intensity ultraviolet light to control the resin curing rate.

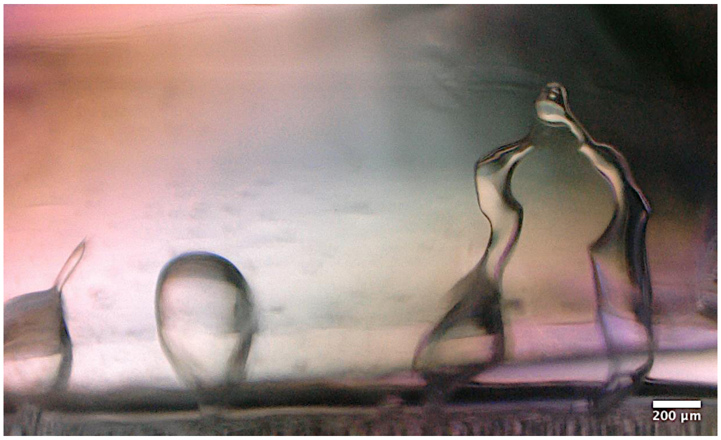

Figure S10: Loop ice structures (from the Figure S7) were embedded in UV-curable resin. However, here the resin was cured using continuous high ultraviolet light intensity. This melted the ice templates, resulting in a tremendous mismatch between the intended and actual channel geometry. This highlights the critical need for using ramped ultraviolet light intensity during the curing step.

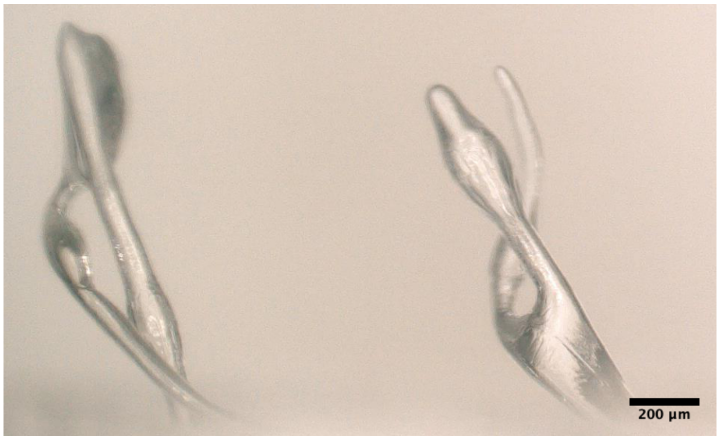

Figure S11: Helix ice structures were embedded in UV-curable resin. The resin was cured using continuous high ultraviolet light intensity. This melted the ice templates, resulting in a tremendous mismatch between the intended and actual channel geometry.

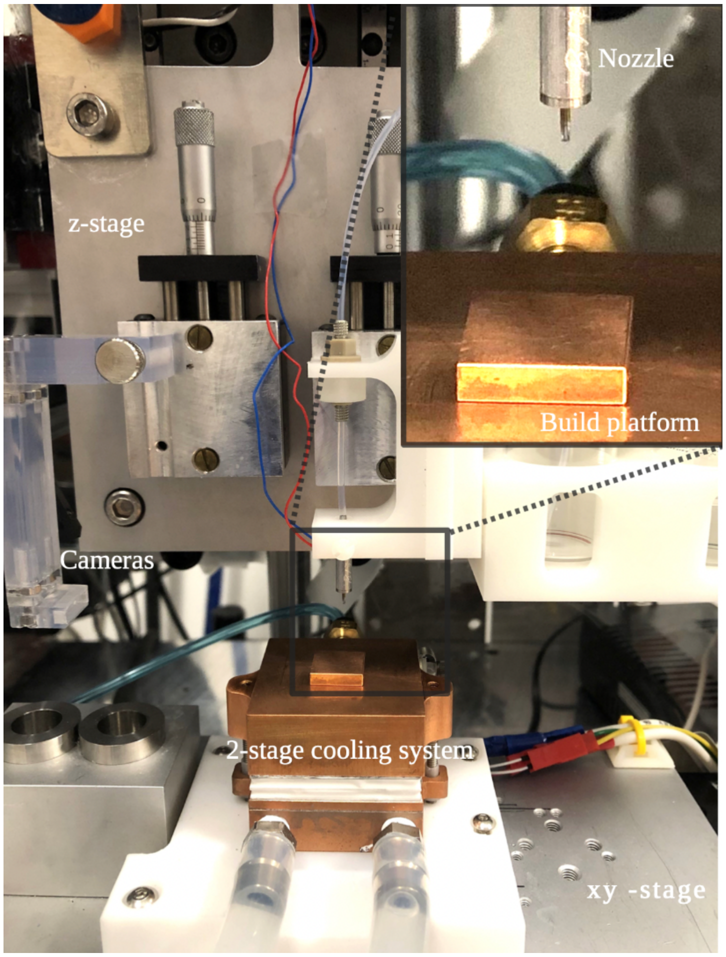

Figure S12: The custom-built 3D printing system and its principal components, including the cooling system,motion stages and piezoelectric nozzle.

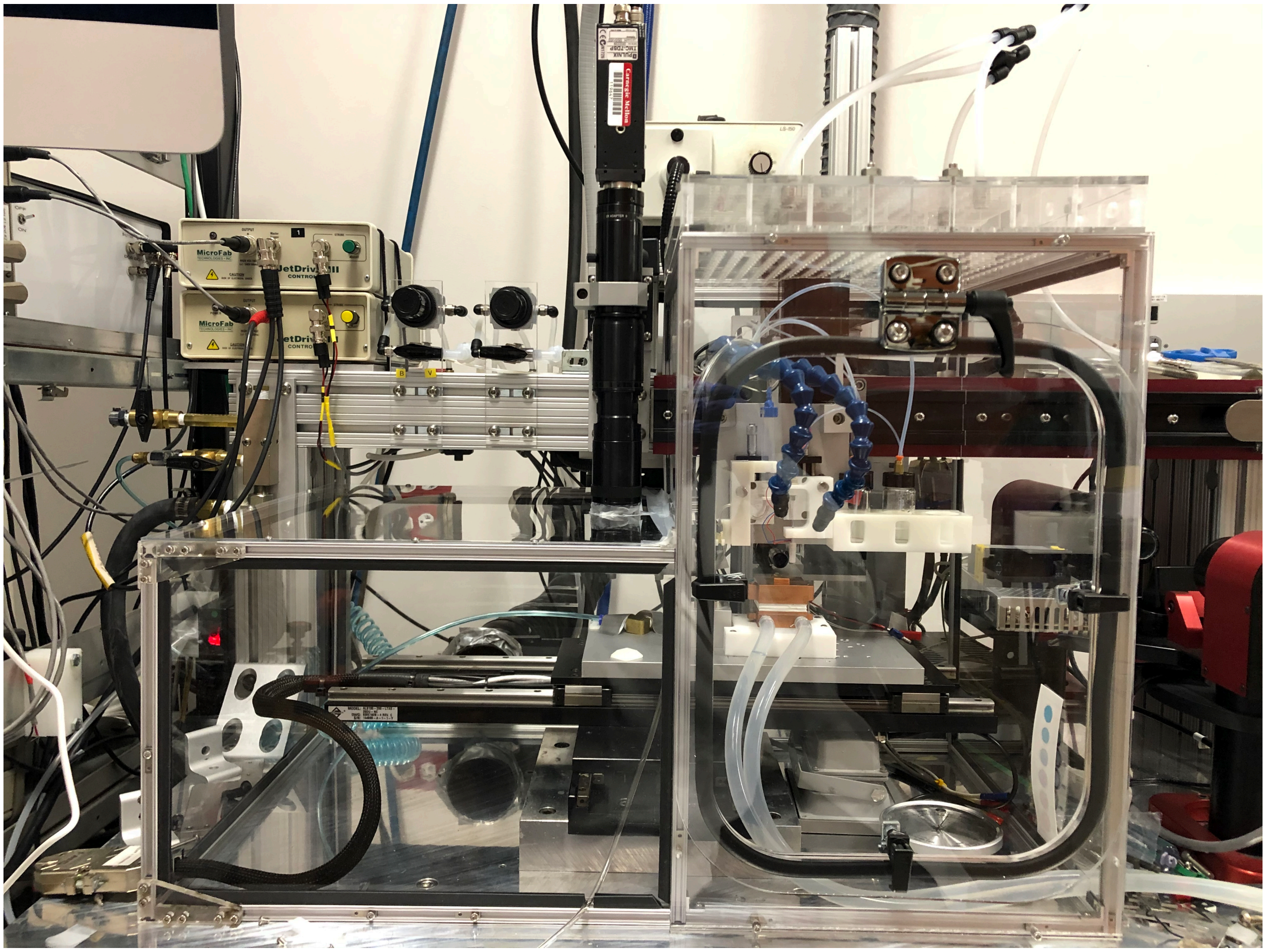

Figure S13: Custom-built enclosure to control local environmental conditions around the print platform. We use a dry nitrogen purge to remove moisture from the enclosure.

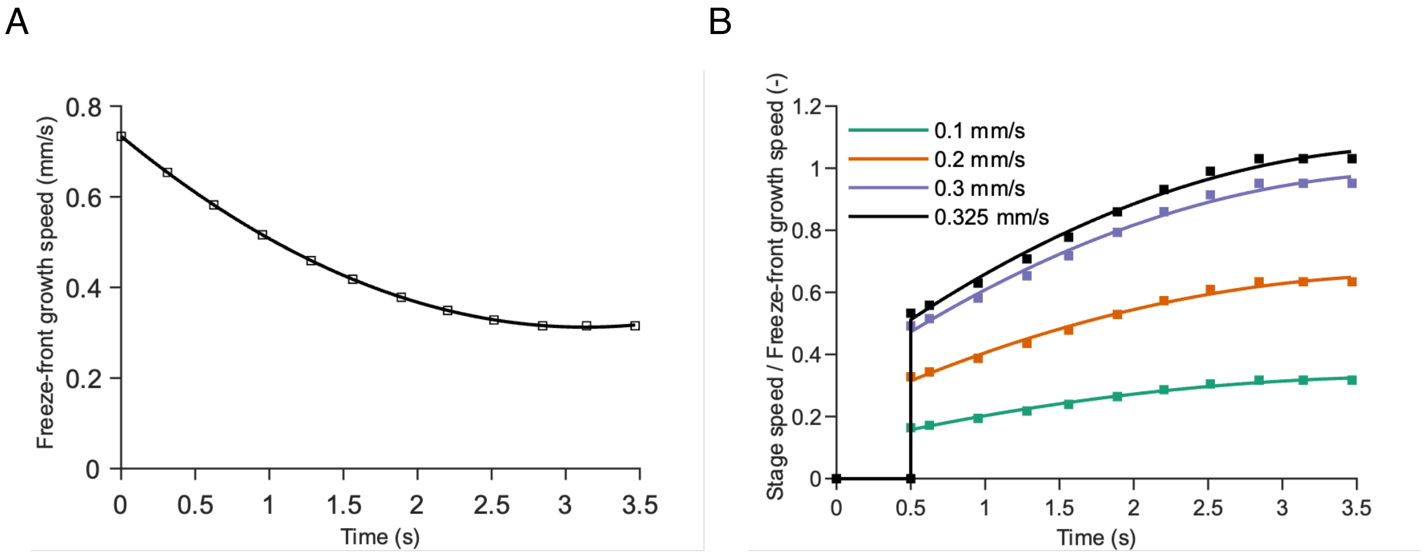

Figure S14: (A) Freeze-front growth speed as a function of time for the pillars printed in Figure 3(A). (B) Ratio of stage transitional speed to the freeze-front growth speed corresponding to the stage translation speeds 0.1, 0.2, 0.3 and 0.325 mm/s. Stage motion starts at 0.5s for all three cases. For stage translation speed of 0.325 mm/s the ratio exceeds 1, resulting in the incoming droplet to miss impinging on the existing pillar and starts a new pillar (Figure 1(B)).

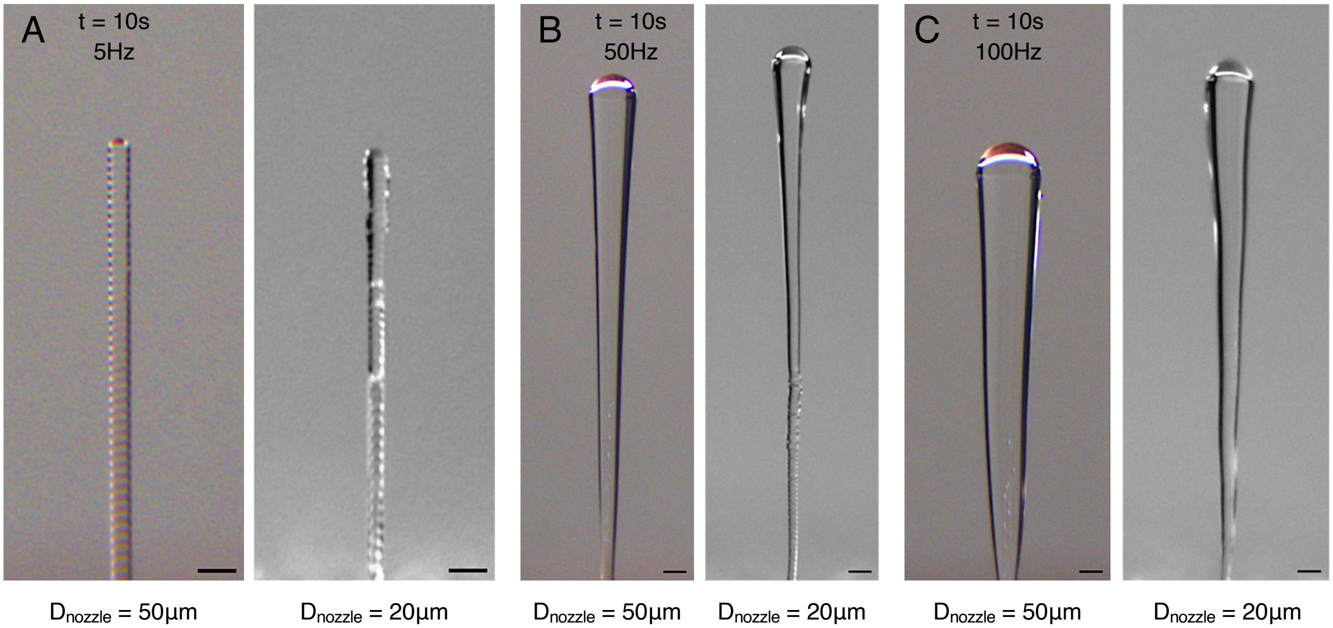

Figure S15: Printing ice columns with an inkjet nozzle with diameter = 20 µm. (A-C) Printing droplets at 5, 50 and 100 Hz without moving the stage. Figures on the left in each part illustrate geometries printed using the 50 µm diameter nozzle using the same print code. With the 20 µm nozzle layered deposition is observed for the entire 5Hz column and half way through the 50Hz printed column. Since the smaller droplets freeze quicker, printing smooth geometries requires a higher droplet deposition frequency. All scale bars are 100 µm.

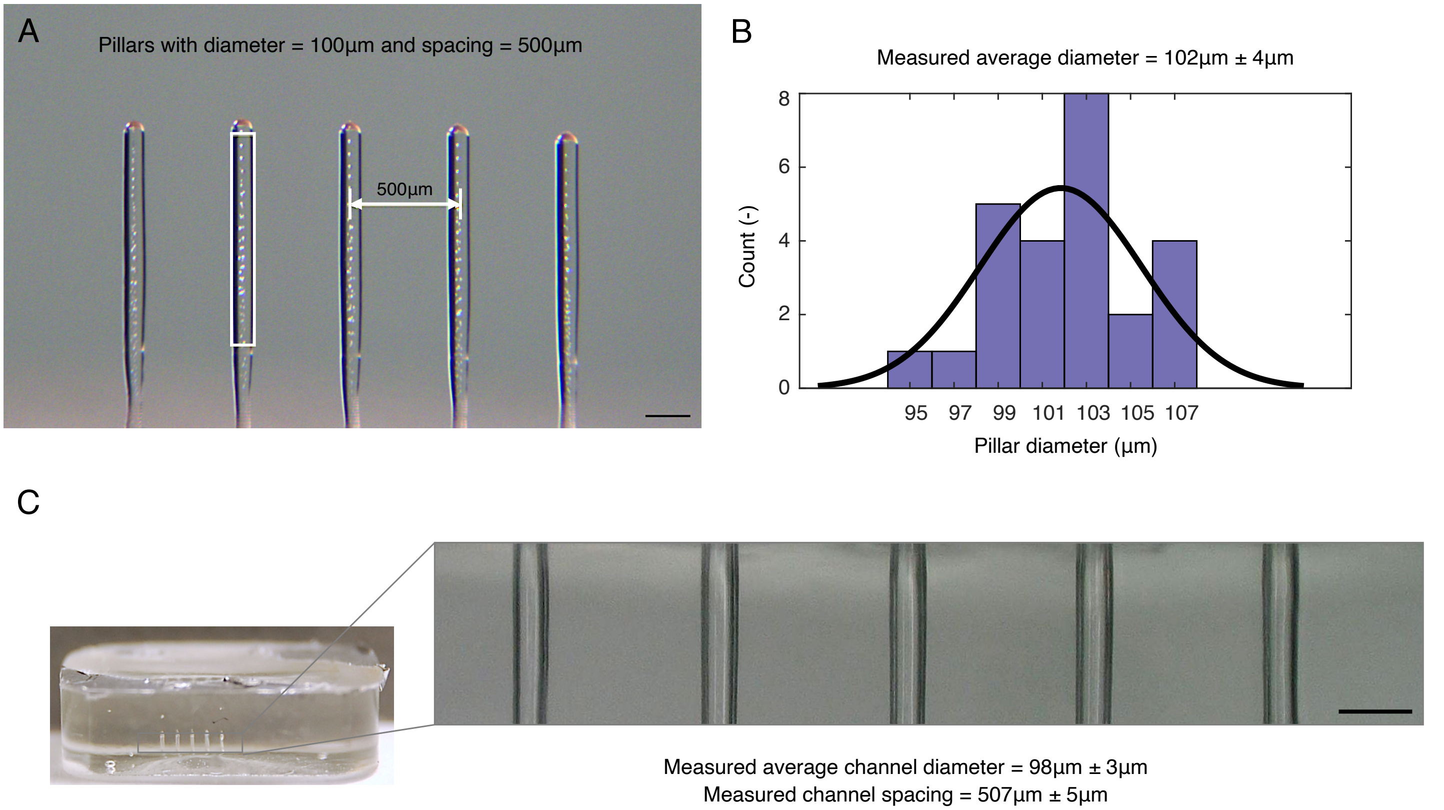

Figure S16: Repeatability of the ice printing and reverse molding process. (A) 25 (5 sets of 5) constant diameter ice pillars spaced 500 μm apart were printed. The average pillar diameter is measured for each pillar by fitting a rectangle to the constant diameter region as shown on the second pillar. (B) Histogram showing the distribution of the pillar diameters. Pillar diameters are closely spread around the mean. The black line illustrates a normal distribution fitted to the same data. (C) To quantify the repeatability of the reverse molding step, 5 printed pillars were used to make channels in UV-curable resin. The reverse molding step is found to closely preserve the diameter and spacing of the ice templates. All scale bars are 200 μm.

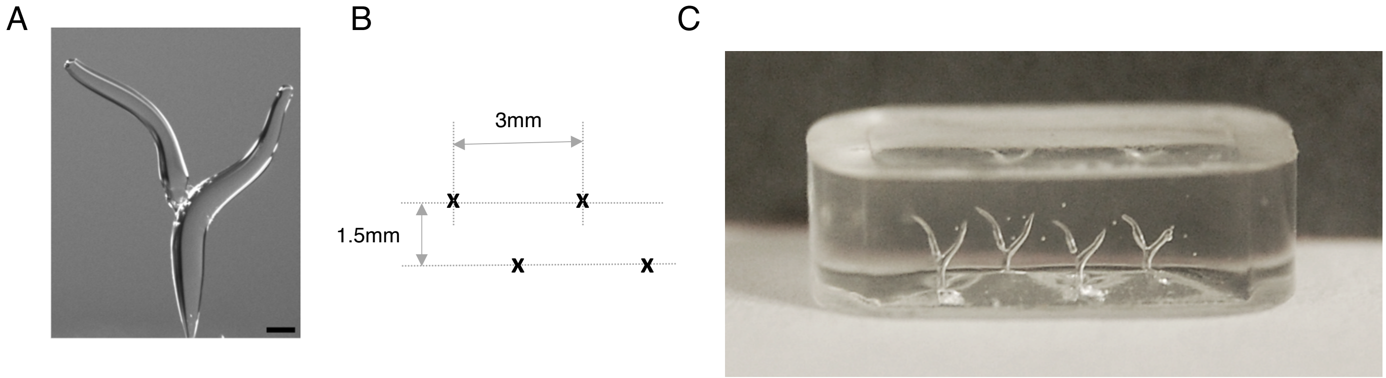

Figure S17: Filling the resin with more structures. 4 repeats of the branched structure shown in (A) were printed in a 2x2 grid with spacing as shown in (B). The resulting ice structures were used to create the geometry in resin as shown in (C).

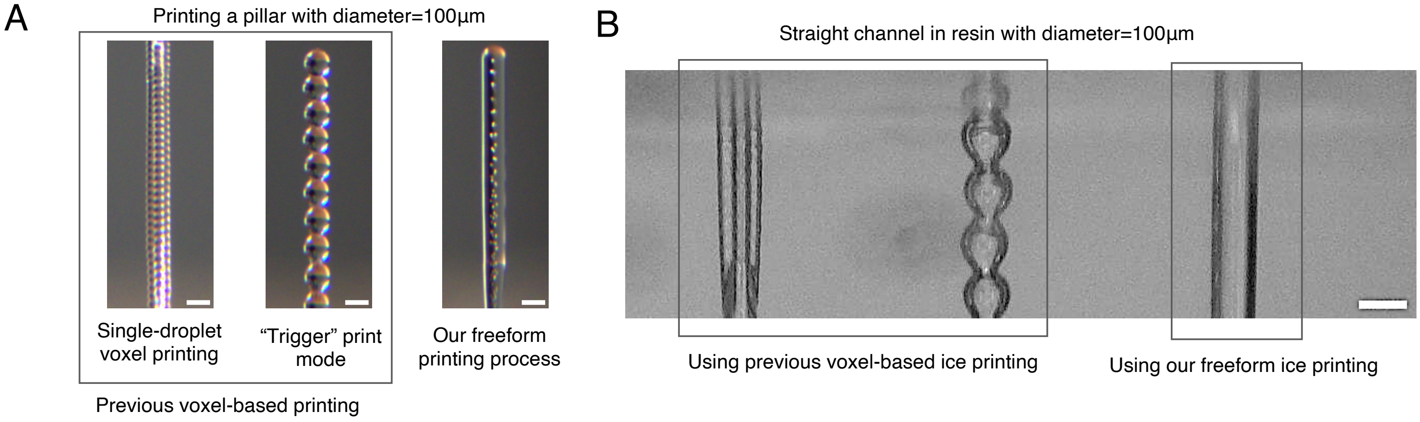

Figure S18: Fabricating constant diameter channels in a UV-curable resin using voxel-based vs freeform printing (A) Ice structures printed using Zheng et al.'s voxel-based approach (left) vs our freeform approach (right). The single-droplet voxel-based columns are printed using stacks of droplets and have non-circular cross-sections. The pillars printed using the large-voxel "trigger mode" show distinct undulations along the pillar length. In contrast, our freeform printed column has smooth walls and constant cross-section. (B) Longitudinal cross-sections of channels fabricated by dipping these ice templates into a UV-curable resin. The smooth walls and controllable diameter of the channels created from the freeform printed ice make them more favorable for many biological and engineering applications. All scale bars are 100 µm.

Movies

Movie S1: Water droplets deposited onto the cold build stage at 200 Hz. After the pillar was approximately 1.5mm tall, the stage was moved 250 µm in a single step at t=0.081s. This resulted in a gradual spatial turning of the freeze front. The red arrows indicate the current position of the freeze front in each frame.

Movie S2: Water droplets deposited onto the cold build stage at 5 Hz. Each individual droplet freezes before the next one is deposited, resulting in a layered geometry.

Movie S3: Water droplets deposited onto the cold build stage at 100 Hz. The pillar widens with increasing height because of a reduction in the rate of heat transfer at the freezing interface.

Movie S4-6: Ice printing columns with 40, 60 and 80 deg overhang respectively.At this size scale, surface tension forces tightly adhere the liquid cap onto the ice, enabling us to print steep overhang angles.

Movie S7-9: Ice printing the geometries illustrated in Figure 4, a helix with an independent central pillar, a branched tree and an octopus respectively.

Printing overhang geometries

To successfully print ice geometries with steep overhang features, the hemispherical liquid water cap needs to stay pinned to the ice pillar. The gravitational force pulling the droplet down is counteracted by surface tension forces at the ice-water interface. The relative importance of these two forces can be calculated using the Bond number ($Bo = \rho g L^2/\sigma$). For ice geometries with 50-400 µm features, $Bo \sim \mathcal{O}(10^{-4})$, indicating that surface tension dominates over gravitational force. This enables printing of steep overhang shapes without the liquid cap sliding off the ice pillar.

Characterizing vertical pillar growth

For a vertically growing ice pillar the total water volume of the deposited pillar was calculated by multiplying ice volume of the pillar with the ratio of water to ice densities. The pillar ice volume was estimated by integrating the cross-sectional area along the pillar height. Pillar cross-sectional area was calculated from the pillar width measured at regular intervals along the pillar height. Total deposited water volume at any instant was calculated as the product of droplet volume and total number of droplets deposited until that instant. The freezing rate was then estimated as the time derivative of the water volume of the growing ice pillar. Heat flux at the freezing interface was estimated from the freezing rate and latent heat of freezing for water.

Identification of the freeze front from camera images

To controllably print complex ice geometries with our freeform method we tracked the freezing front location, width and heading angle. The freezing front was identified by the slight color difference between the liquid water cap and the frozen ice. As described in the main text, an off-axis deposition of water on a growing ice pillar starts to turn the freezing front in the direction of the deposition. In **Movie S1**, water droplets were deposited onto the cold build stage at a fixed 200 Hz frequency. After the pillar was approximately 1.5 mm tall, we started depositing water off-axis by moving the nozzle by 250 µm in a single step at t=0.081s. This resulted in a gradual turning of the freeze front. The red arrows indicate the current position of the freeze front in each frame. **Figure S1** shows three frames from the video, with the freezing front location marked. Note that individual water droplets are not visible in the video because of the considerably slower frame rate of the video (approx. 12 fps) in relation to the droplet velocity (approx. 2 m/s).

Comparison between voxel-based methods and 3D-ICE

To further illustrate the distinct advantage of the 3D-ICE process over the voxel-based methods when printing geometries with smooth surfaces, we printed a pillar with approximately 100 µm diameter using the 50 µm diameter water droplets. Printing this geometry using a voxel-based approach is challenging because the desired feature size (i.e., the pillar diameter) is commensurate with the voxel size (i.e., the droplet diameter), resulting in a very coarse discretization. We printed the pillars using the two distinct voxel-based strategies [7, 8] and with 3D-ICE. First, each individual droplet is treated as a single voxel by performing the printing at a low frequency (2 Hz). Since the voxel is a discrete unit, features can only be printed by combining individual voxels. Using this technique, the best possible approximation of a circular cross-section is the deposition of three individual voxels in a triangular configuration, resulting in a non-circular cross-section (Figure S18 (A)). Next, we fabricated the pillar using larger voxels printed by using high frequency (1500 Hz) bursts of droplet ejection (the "trigger mode" [7]) and pausing deposition between printing of subsequent voxels to allow each voxel to completely freeze before the next one is deposited. Although the width of the resulting ice pillars (Figure S18 (A)) matches the intended pillar width, the larger voxels create a distinct undulation in the pillar diameter along the

length of the pillar, deviating from the intended geometry. These wider voxels are also taller, further reducing the fidelity of the process to capture sharp gradients in channel diameter. Finally, the same pillar printed using our freeform process is also shown in Figure S18 (A). Figure S18 (B) illustrates the longitudinal cross-section of channels fabricated in resin corresponding to these three printed geometries using our reverse-molding process (described in greater detail in subsequent sections). Unlike the channels fabricated from the voxel-printed ice geometries, our freeform process produces high-fidelity geometries (a constant circular cross-section with 100 µm diameter) with smooth surfaces."
